# Bilateral Cavernous Sinus Thrombosis in Presumed COVID-19 Infection

**DOI:** 10.7759/cureus.31986

**Published:** 2022-11-28

**Authors:** Stephen A LoBue, Royce Park, Richard Giovane, John DeLury, Nickisa Hodgson

**Affiliations:** 1 Ophthalmology, State University of New York Downstate Medical Center, New York, USA; 2 Family Medicine, University of Alabama, Tuscaloosa, USA; 3 Infectious Disease, State University of New York Downstate Medical Center, New York, USA

**Keywords:** severe acute respiratory syndrome coronavirus 2 (sars-cov-2), bilateral cavernous sinus thrombosis, septic cavernous sinus thrombosis, orbital cellulitis, covid-19

## Abstract

Cavernous sinus thrombosis (CST) is a rare life-threatening condition where a blood clot develops within the cavernous sinus secondary to various etiologies, ranging from infection to aseptic causes (e.g., trauma or surgery). The dural sinuses and the cerebral veins have no valves, which allow retrograde blood flow according to pressure gradients. As a result, cavernous sinuses are vulnerable to septic thrombosis from infection at various sites including sphenoid and ethmoid sinuses. Less commonly, infections of the face, ears, nose, tonsils, soft palate, and teeth may lead to CST if treatment is delayed. Clinical findings of CST extending to the opposite cavernous sinus typically requires 24-48 hours after the initial presentation of orbital signs. However, we present a patient with facial and orbital cellulitis that was immediately treated with high-dose IV antibiotics within one hour of presentation and IV heparin six hours after admission and CST diagnosis. However, the patient developed a rapid progression of bilateral CST within six hours, unresponsive to treatment. Although facial cellulitis may lead to septic CST if untreated, the rapid progression of bilateral CST in the setting of acute hypoxic respiratory failure, renal failure, and coagulation abnormalities suggests a possible underlying infection and complications similar to severe acute respiratory syndrome coronavirus 2 (SARS-CoV-2) infection.

## Introduction

Since the current global outbreak of severe acute respiratory syndrome coronavirus 2 (SARS-CoV-2) in December 2019, over 628 million cases and 6.5 million deaths have been reported worldwide [1]. The coronavirus disease 2019 (COVID-19) pandemic has been described as the worst global public health crisis in modern history as it continues to wreak social and economic disruption worldwide. In an effort to mitigate both spread and mortality, there has been an impressive and unprecedented global research response into the clinical characteristics and outcomes of SARS-CoV-2 infection. The reported acute complications of COVID-19 include acute respiratory distress syndrome, cardiac and kidney injury, and shock [2]. Hypercoagulability, pulmonary and venous thromboembolism, and cerebral venous sinus thrombosis have also been observed in severe cases [3,4]. Herein, we report a case of a patient who presented with orbital and facial cellulitis that rapidly progressed to bilateral cavernous sinus thrombosis within six hours of presentation. The patient’s clinical deterioration occurred in the context of septic shock, acute hypoxic respiratory failure, renal failure, coagulopathy, and multifocal pneumonia, which was unresponsive to IV antibiotics and anticoagulants, all complications consistent with SARS-CoV-2 infection.

## Case presentation

A 62-year-old male with a history of well-controlled hypertension and depression presented to the Kings County Hospital Center emergency room in March 2020 with several days of right-sided eyelid swelling and forehead pain. The patient denied any vision changes, headaches, fevers, chills, nausea, dizziness, or recent trauma or bug bite leading up to the presentation.

**Figure 1:**
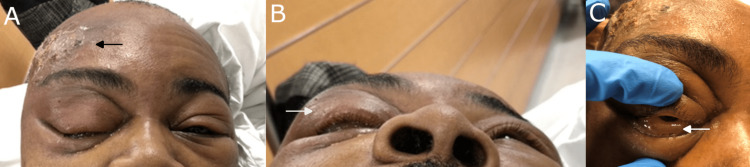
Initial clinical presentation (A) Crusted, scaling erythematous lesion on the right forehead of the patient on initial presentation (arrow). (B) Right upper and lower lid edema and proptosis on worm’s-eye view (arrow). (C) Severe chemosis of the right eye (arrow).

On external examination, the patient had a crusted, scaling erythematous lesion on the right forehead. No active discharge was present (Figure 1A). The patient was alert and oriented to person, place, and time. Vitals were stable with a blood pressure of 130/80 mmHg, respiratory rate of 16 breaths per minute, heart rate of 85 beats per minute, temperature of 98.9°F, and pulse oxygenation of 99%.

The patient’s visual acuity was 20/30 in the right eye (OD) and 20/20 in the left eye (OS). The patient’s intraocular pressures (IOP) were 34 OD and 15 OS. There was a significant limitation of extraocular movements in all directions OD with associated mild pain. There was severe right upper and lower lid edema and erythema, proptosis (Figure 1B), and 360 degrees of chemosis (Figure 1C).

**Figure 2:**
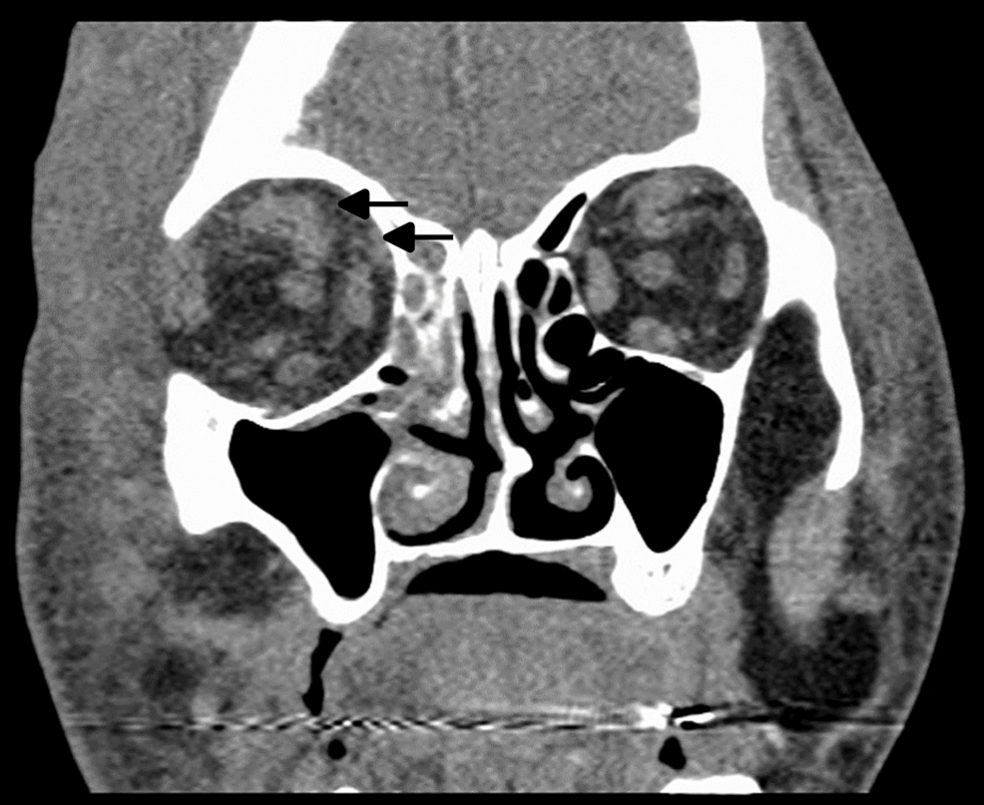
Computed tomography of the orbit CT of the orbits demonstrating stranding and infiltration of the intraorbital fat surrounding the right superior rectus/superior ophthalmic vein and medial to the medial rectus suspicious for orbital cellulitis (black arrows). Note the facial cellulitis and that the right is greater than the left. CT: computed tomography

Initial laboratory results demonstrated leukocytosis (25.77 K/uL) with neutrophilic predominance (88.1%) and left shift (16% bands), elevated creatinine of 1.14 mg/dL, lactate of 2.43 mmol/L, thrombocytosis (platelet: 837 × 10^9^/L), prothrombin time (PT) of 18.4 seconds, and international normalized ratio (INR) of 1.6. Computed tomography (CT) of the orbits showed right orbital fat stranding and diffuse facial cellulitis (Figure 2). The patient was started on IV vancomycin and piperacillin-tazobactam for the management of suspected right orbital cellulitis.

**Figure 3:**
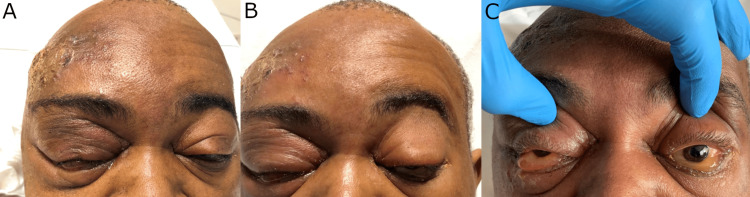
Clinical reevaluation (A) Reevaluation five hours after the initial examination demonstrating a progression of right-sided orbital erythema with chemosis. In the left eye, worsening ptosis and limitation of extraocular movements were newly appreciated on reexamination at this time. (B and C) Reevaluation about 12 hours after presentation showing worsening of ptosis and chemosis of both eyes with bilateral frozen globes.

Ophthalmic reevaluation five hours later revealed worsening right-sided orbital erythema and chemosis, decreased visual acuity to 20/100 OD and 20/40 OS, new ptosis, and restriction of extraocular movements in the left eye (Figure 3).

**Figure 4:**
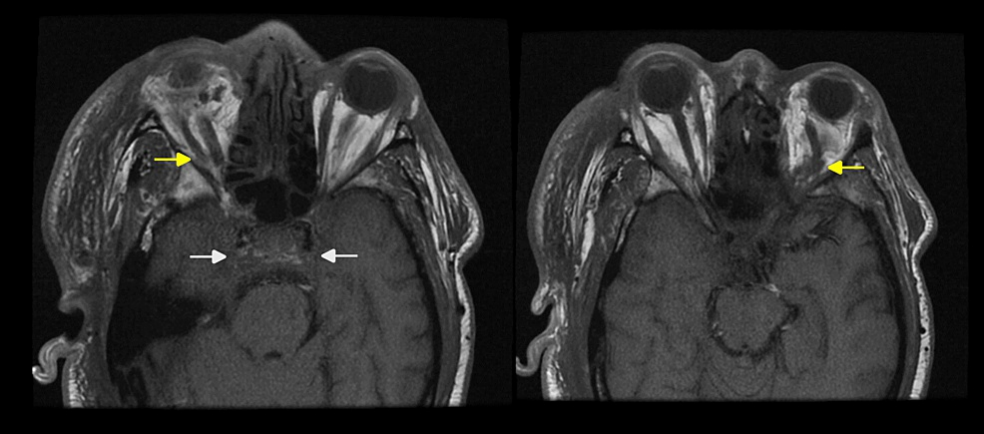
Axial T1 weighted MRI brain and orbits Axial T1-weighted MRI of the brain and orbits with contrast depicting bilateral cavernous sinus thrombosis (white arrows), bilateral superior ophthalmic vein thrombosis (yellow arrows), bilateral orbital cellulitis and proptosis with the right greater than the left, and right-sided pachymeningeal thickening and enhancement concerning for meningitis. MRI: magnetic resonance imaging

Magnetic resonance imaging (MRI) and magnetic resonance venography (MRV) of the brain and orbits confirmed superior ophthalmic vein thrombosis, bilateral cavernous sinus thrombosis, and right-sided pachymeningeal thickening, which was concerning for meningitis (Figure 4).

**Figure 5:**
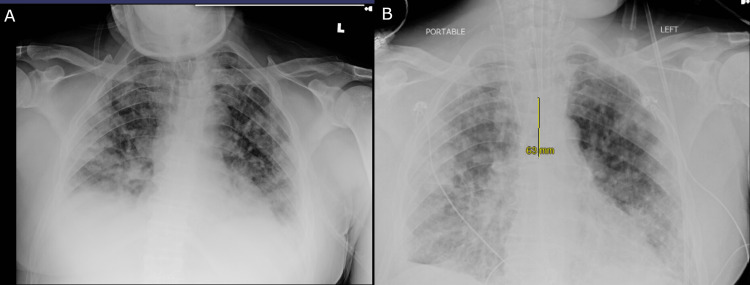
Anterior/posterior chest X-rays AP chest X-rays demonstrated bilateral hazy airspace disease with probable underlying pleural effusions, suggestive of multifocal pneumonia. Films were taken before (A) and after (B) endotracheal intubation. AP: anterior/posterior

Within 24 hours of admission, the patient’s mental and respiratory status promptly deteriorated. Chest X-ray revealed bilateral hazy airspace disease with underlying pleural effusions (Figure 5), which was concerning for multifocal pneumonia.

The patient was emergently intubated due to the potential of acute hypoxic respiratory failure. Nasopharyngeal swab testing with the cobas® SARS-CoV-2 test (Roche Diagnostics International AG, Rotkreuz, Switzerland) was negative. Laboratory evaluation showed lymphopenia (lymphocytes: 0.24 K/uL), severely elevated serum D-dimer of 7,585 ng/mL, activated partial thromboplastin time (aPTT) of >120 seconds, PT of 38.5 seconds, INR of 3.7, and fibrinogen of 827 mg/dL. Blood cultures were positive for methicillin-sensitive *Staphylococcus aureus*. The patient developed septic shock requiring vasopressors and renal failure requiring continuous veno-venous hemofiltration. The patient was also started on IV heparin. Nasopharyngeal swab testing five days later with BioReference COVID-19 PCR assay was also negative. COVID-19 antibody testing was not yet available during this time. Additionally, the patient’s respiratory viral panel, transthoracic echocardiography, urine culture, and clostridium difficile immunoassay were all negative. The patient had a prolonged ICU course, remaining unresponsive with no improvement in his clinical condition. The patient’s next of kin elected for pursuit of comfort care measures, and the patient died on hospital day 14.

## Discussion

Cavernous sinus thrombosis (CST) is a rare and potentially fatal diagnosis. The cavernous sinuses are positioned around the sella turcica, which surrounds the pituitary gland and is in close proximity to cranial nerves III, IV, V, and VI and the horizontal segment of the internal carotid artery. The dural sinuses and the cerebral veins have no valves, which allow retrograde blood flow according to pressure gradients. As a result, cavernous sinuses are vulnerable to septic thrombosis from infection at various sites including sinusitis, specifically the sphenoid and ethmoid sinuses. With the use of broad-spectrum IV antibiotics, primary source infections of the face, ears, nose, tonsils, soft palate, and teeth have been less common [5]. Common clinical findings of CST include fever, ptosis, proptosis, chemosis, and external ophthalmoplegia (paralysis of the extraocular muscles). Clinical findings of CST extending to the opposite cavernous sinus typically requires 24-48 hours after the initial presentation of orbital signs [5]. Mortality from CST has decreased from 80% to 100% in the pre-antibiotic era to 20% to 30% since 1940 [5]. Management of patients with CST includes treating the underlying infection with an extended course of high-dose IV antibiotics. Full anticoagulation using heparin or warfarin may also be beneficial in select patients as anticoagulant therapy begun within seven days of hospitalization may reduce morbidity rates in survivors [6].

Nevertheless, our patient presented with facial and orbital cellulitis, which was immediately treated with high-dose IV antibiotics within one hour of presentation and IV heparin six hours after admission and CST diagnosis. However, the patient developed a rapid progression of bilateral CST within six hours, unresponsive to treatment. Due to the clinical, laboratory, and radiographic characteristics of this case, we hypothesize that COVID-19 may have precipitated a prothrombogenic state, known as COVID-19-associated coagulopathy (CAC) [7].

CAC is characterized by coagulation disorders that affect various tissues, resulting in skin purpura, myocardial infarction, and neurological dysfunction [8]. Evidence supports the concept that CAC involves complex interactions between vascular endothelium, coagulation and fibrinolytic pathways, and the innate immune response [8]. Circulating microthrombi or macrothrombi can lead to multi-organ injury or failure [8]. Thus, CAC could have potentially accelerated the rapid development of a bilateral cavernous sinus thrombosis within 12 hours in an otherwise immunocompetent patient with comorbidities limited to well-controlled hypertension and depression. Although there have been cases of cerebral venous sinus thrombosis in COVID-19 patients, bilateral cavernous sinus thrombosis in the setting of clinical characteristics suggestive of COVID-19 has not been previously described [9].

Several published neuro-ophthalmic case reports have described nasopharyngeal swabs negative for SARS-CoV-2, but serology specimens positive for IgG antibodies. For example, in a case of secondary pseudotumor cerebri syndrome, a patient tested negative for SARS-CoV-2 on admission but tested positive on COVID-19 IgG qualitative testing following a 14-day hospital course complicated by respiratory failure requiring intubation, septic shock, and pulmonary radiographic findings consistent with COVID-19 [10]. In another case, a patient tested negative for SARS-CoV-2 on admission via nasopharyngeal swab with extensive pulmonary disease; two weeks later, the patient developed altered mental status, elevated D-dimer, and cerebral and pulmonary thrombosis [11]. A nasopharyngeal sampling at this time was negative, but the serologic analysis was positive for 2019-nCoV IgG antibodies [11]. The discrepancy in testing results over time suggests that CAC is a late complication of severe COVID-19, likely associated with a prolonged inflammatory reaction [9].

Unfortunately, serology testing for antibodies was not available in New York State until late April 2020, and retroactive testing is unavailable at our medical center as all blood samples are discarded after seven days. Additionally, the timing of this case supported our clinical suspicion of SARS-CoV-2 infection. The patient presented to our medical center in New York City in mid-March, 10 days after the governor declared a state of emergency and seven days after the World Health Organization (WHO) declared the coronavirus outbreak a pandemic.

While it may be speculative to presume that our patient was infected with SARS-CoV-2 despite negative nasopharyngeal specimens and the absence of serology testing, the patient’s acute hypoxic respiratory failure, chest X-ray findings of multifocal pneumonia, renal failure, and coagulation abnormalities were all consistent with severe SARS-CoV-2 infection.

The increased hypercoagulable state of our patient also supports a COVID-19 infection. Compared with diseases caused by other common respiratory viral infections, patients with COVID-19 have a higher frequency and severity of coagulable events associated with elevated levels of D-dimer, C-reactive protein, and fibrinogen [12].

It is also possible that elevated D-dimer levels in patients with COVID-19 are accompanied by only occasional prolongation of the prothrombin time and activated partial thromboplastin time in combination with elevation to platelet counts as seen in our patient [13]. Furthermore, CAC has been known to affect the central nervous system (CNS). In serial histological analyses of 100 COVID-19-positive autopsies, 58 brains revealed widespread microthrombi and microinfarcts in the neocortex [14].

## Conclusions

There is an increasingly recognized association between COVID-19 and hypercoagulability known as CAC. Due to the severe prothrombotic state, circulating microthrombi or macrothrombi can lead to multi-organ injury or failure including the CNS. Our patient presented with facial and orbital cellulitis, which was immediately treated with high-dose IV antibiotics within one hour of presentation and IV heparin six hours after admission and CST diagnosis. However, the patient developed a rapid progression of bilateral CST, unresponsive to treatment. Although facial cellulitis may lead to septic CST if untreated, the rapid progression of bilateral CST in the setting of acute hypoxic respiratory failure, renal failure, and coagulation abnormalities suggests a possible infection and complications similar to severe SARS-CoV-2 infection. Thus, more investigation is required to better understand the mechanisms and risk factors to better treat and prevent COVID-19-associated coagulopathy.
